# Anti-Drug Antibodies in the Biological Therapy of Autoimmune Rheumatic Diseases

**DOI:** 10.3390/jcm12093271

**Published:** 2023-05-04

**Authors:** Oscar Pizano-Martinez, Edgar Mendieta-Condado, Mónica Vázquez-Del Mercado, Erika Aurora Martínez-García, Efrain Chavarria-Avila, Daniel Ortuño-Sahagún, Ana Laura Márquez-Aguirre

**Affiliations:** 1 Instituto de Investigación en Reumatología y del Sistema Músculo-Esquelético (IIRSME), Centro Universitario de Ciencias de la Salud, Universidad de Guadalajara, Guadalajara 44340, JAL, Mexico; oscar.pizano@academicos.udg.mx (O.P.-M.);; 2 Departamento de Morfología, Centro Universitario de Ciencias de la Salud, Universidad de Guadalajara, Guadalajara 44340, JAL, Mexico; 3 Cuerpo Académico UDG-CA-703, Centro Universitario de Ciencias de la Salud, Universidad de Guadalajara, Guadalajara 44340, JAL, Mexico; 4 Laboratorio Estatal de Salud Pública (LESP), Secretaría de Salud Jalisco, Zapopan 46170, JAL, Mexico; 5 Departamento de Biología Molecular y Genómica, Centro Universitario de Ciencias de la Salud, Universidad de Guadalajara, Guadalajara 44340, JAL, Mexico; 6 Departamento de Fisiología, Centro Universitario de Ciencias de la Salud, Universidad de Guadalajara, Guadalajara 44340, JAL, Mexico; 7 Departamento de Disciplinas Filosófico, Metodológicas e Instrumentales, Centro Universitario de Ciencias de la Salud, Universidad de Guadalajara, Guadalajara 44340, JAL, Mexico; 8 Instituto de Investigación en Ciencias Biomédicas (IICB), Centro Universitario de Ciencias de la Salud, Universidad de Guadalajara, Guadalajara 44340, JAL, Mexico; 9 Unidad de Biotecnología Médica y Farmacéutica, Centro de Investigación y Asistencia en Tecnología y Diseño del Estado de Jalisco A.C. (CIATEJ), Guadalajara 44270, JAL, Mexico

**Keywords:** autoimmune rheumatic diseases, anti-drug antibodies, biological agents, immunogenicity

## Abstract

Autoimmune rheumatic diseases are a cluster of heterogeneous disorders that share some clinical symptoms such as pain, tissue damage, immune deregulation, and the presence of inflammatory mediators. Biologic disease-modifying antirheumatic drugs are some of the most effective treatments for rheumatic diseases. However, their molecular and pharmacological complexity makes them potentially immunogenic and capable of inducing the development of anti-drug antibodies. TNF inhibitors appear to be the main contributors to immunogenicity because they are widely used, especially in rheumatoid arthritis. Immunogenicity response on these treatments is crucial since the appearance of ADAs has consequences in terms of safety and efficacy. Therefore, this review proposes an overview of the immunogenicity of biological agents used in autoimmune rheumatic diseases highlighting the prevalence of anti-drug antibodies.

## 1. Introduction

Autoimmune rheumatic diseases (ARDs) are a cluster of heterogeneous disorders that share some clinical symptoms such as pain, tissue damage, immune deregulation, and the presence of inflammatory mediators. Disease-modifying antirheumatic drugs (DMARDs) are immunosuppressive and immunomodulatory agents classified as either synthetic (sDMARDs)—conventional synthetic (csDMARDs) and targeted synthetic DMARDs (tsDMARDs)—or biologic agents (bDMARDs). They were introduced in the late 1990s and are usually prescribed after the failure of conventional therapy [1,2].

bDMARDs have become an important component of therapeutic strategies for many ARDs. However, these biologics are protein drugs that tend to induce an immune response against themselves (immunogenicity), particularly the formation of anti-drug antibodies (ADAs), which can have clinical consequences that depend on the type of the ADAs (neutralizing or non-neutralizing antibodies) and the resulting immune complexes that may form with the biologic agent [3,4,5].

Many factors may influence the development of an immune response against a therapeutic protein and could be classified as patient- or drug-related factors. Patient- and disease-related factors stand out in the context of ARDs, as patients with activated immune systems may be more prone to immune responses to therapeutic proteins. However, there is evidence that a combination with immunosuppressive agents (e.g., methotrexate, MTX) during biologic therapy reduces the risk of ADAs production and may induce tolerance to biologic therapy in patients with ARDs [6,7,8]. For this reason, the duration of dual immunosuppressive therapy necessary to ensure tolerance to the biological component should be studied.

On the other hand, although the procedures for immunogenicity testing of therapeutic protein products and detection of ADAs have been well established by the US Food and Drug Administration (FDA) [9] and the European Medicines Agency (EMA) [10], there are many discrepancies in assessing and reporting of clinical immunogenicity. This could be due to the fact that the detection of ADAs depends on many factors, including the timing of sample collection relative to dosing, the duration of treatment, and the assay used [11].

In this review, we summarize the data related to the biological agents approved for ARDs, factors influencing their immunogenicity, and immunogenic reports, focusing on the frequency of ADAs in clinical trials for ARDs and their potential clinical consequences.

## 2. Autoimmune Rheumatic Diseases and Biological Therapy

### 2.1. ARDs Classification

The exact etiology of ARDs is poorly understood but is thought to be due to a complex interaction of multiple factors. Dysregulation of the immune system may precede clinical symptoms by years [12]. Autoimmune diseases can be classified according to several criteria. One of them is the site of an autoimmune attack. Based on this criterion, these disorders can be divided into systemic and organ-specific. Systemic autoimmune diseases or rheumatic autoimmune diseases (SARDs) affect two or more organs and tissues, such as systemic lupus erythematosus (SLE), multiple sclerosis (MS), or rheumatoid arthritis (RA). In organ-specific or non-systemic autoimmune rheumatic diseases (non-SARDs), a specific organ or tissue is preferentially attacked by the patient’s immune system, such as in psoriasis (Ps) or ankylosing spondylitis (SpA), according to the American College of Rheumatology (ACR) and European League Against Rheumatism (EULAR) or International Classification Diseases (ICD)-classification by World Health Assembly of World Health Organization in 2019, ICD 11th revision [13,14,15,16].

### 2.2. Biologic Therapy in ARDs

Since pharmacological treatment with csDMARDs, followed by tsDMARDs, and the advent of bDMARDs [17,18,19], therapies for ARDs have evolved considerably. Biological drugs are considered to have an important advantage over synthetic drugs due to their specific molecular target and clinical efficacy. The most frequent adverse event is a mild, transient reaction at the injection site. However, there are also serious, rare, and unpredictable adverse events, such as an increased risk of serious infections. Therefore, the occurrence of adverse drug reactions (ADRs) should be continuously monitored in post-marketing safety studies [20,21].

Biological agents are recombinant therapeutic proteins and have been designed to block different molecular targets. In this review, we show biologics licensed or approved by the FDA or EMA for the treatment of ARDs, including Tumor Necrosis Factor (TNF) agents—also called TNF inhibitors (TNFi), B cell-targeting biologics, a T cell activation inhibitor/co-stimulation modulator, and anti-interleukin (IL)—IL-6, 17A, IL-12/23, IL23p19 and IL-1 agents, Table 1. These biological agents are mostly monoclonal antibodies (mAbs), proteins produced in vitro using recombinant techniques from a single B-lymphocyte clone. Currently, chimeric (65% human), humanized (>90% human), and fully human (100% human) mAbs are available [22,23,24]. On the other hand, the fusion proteins are composed of a receptor portion of the target molecule and the Fc region of the immunoglobulin; the currently available fusion proteins include etanercept (ETA), abatacept (ABT) and anakinra (ANA) [25].

## 3. Immunogenicity of Biologic Agents in ARDs

### 3.1. Immunogenicity Factors and ADAs Development

According to FDA [9], immunogenicity is defined as the propensity of a therapeutic protein product to generate immune responses to itself and related proteins or to induce immunologically related adverse clinical events. Therefore, in the context of therapeutic proteins, immunogenicity is unwanted immune responses, unlike other biotechnological products such as vaccines, where the immune response is desired.

Many factors may influence the development of an immune response against a therapeutic protein, and these can be classified into product or patient- and disease-related. Product-related factors include chemical modifications (e.g., pegylation or fusion proteins), impurities (e.g., degradation products, impurities, and aggregates), formulation interactions between the protein and/or formulation with the primary product packaging (e.g., containers, closures) and the origin and nature of the therapeutic protein (chimeric, humanized or fully human antibodies) [10]. Although the evidence indicates that fully human antibodies are potentially the least immunogenic, some humanized and even fully human sequence-derived antibody molecules still carry immunological risk [3,24].

Patient- and disease-related factors, on the other hand, include genetic factors that modulate the immune response and factors related to a genetic defect. The immune system can detect small differences in the three-dimensional structure between an introduced foreign molecule and a native protein. In this context, recombinant proteins used for therapeutic purposes could represent a neo-antigen. Moreover, treatment-related factors, including concomitant therapies, may either decrease or increase the risk of an immune response to a therapeutic protein [10]. Although patients with activated immune systems (for example, autoimmune diseases) may be more prone to immune responses to therapeutic proteins, there is evidence showing that concomitant use of certain DMARDs may maintain efficacy and prolong drug survival by reducing the immunogenicity of biologics in chronic inflammatory diseases. For example, the use of MTX in TNFi biologic therapies may attenuate the incidence of ADAs in RA, SpA, and CD [6,7,8,26]. This could be due to the fact that the administration of immunosuppressive drugs could induce tolerance by suppressing B cells and consequently reducing ADAs production. However, the mechanisms contributing to immune tolerance are not fully understood.

Other patients- and disease-related factors include dosing schedule and route of administration [10]. As most of the vaccines are given via the subcutaneous (SC) route, the SC route is expected to be more immunogenic than the intravenous (IV) route. However, there is not an extensive number of clinical trials that have directly compared the safety and efficacy of SC and IV dosing regimens for therapeutic proteins or mAbs [27,28].

Consequently, clinical effects of immune responses in subjects are highly variable, ranging from no measurable effect to extremely harmful. The immune response to biological agents, particularly the generation of ADAs, is a key aspect of therapy. ADAs can be generated by (1) a T-cell dependent/independent B cell activation or (2) T-cell independent/dependent B cell activation pathway. In the T-cell dependent pathway, mAbs act as antigens and are internalized by antigen-presenting cells (APCs), processed, and presented to T cells via the cognate interaction between the MHC class II molecules and T-cell receptor. ADAs are generated when a T helper cell (Th) differentiates into a Th1 or Th2 phenotype and, following their cognate interactions with B cells, induces the proliferation of plasma cells (PC) that secrete ADAs. While biologics with multiple epitopes, such as mAbs, can cross-link B cell receptors (BCRs) via the T cell-independent pathway and then directly stimulate B cells to differentiate into PC to produce ADAs [29].

**Figure 1:**
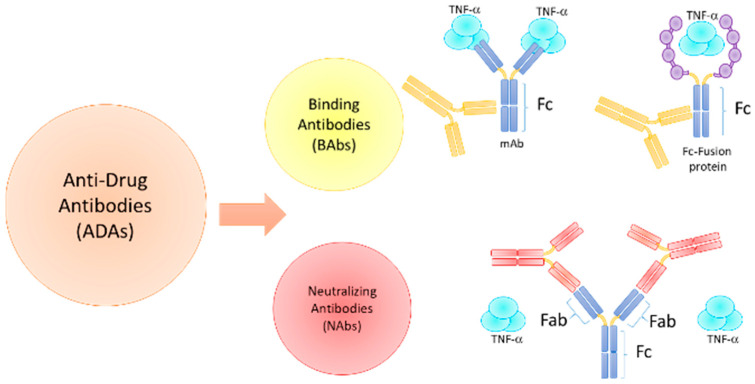
Anti-drug antibodies (ADAs). Two types of ADAs can be distinguished: Non-neutralizing ADA (or binding antibodies, BAbs), -bind to Fc- and does not affect the drug-target interaction but indirectly decrease the drug level by increasing drug clearance via immune complex formation; and neutralizing ADAs (Nabs), which physically interfere with the ability of the drug to bind to its target and it may have a direct negative impact on functional drug level.

Two types of ADAs can be distinguished: non-neutralizing ADAs (or binding antibodies, BAb), which specifically bind the drug but do not affect the drug-target interaction, and neutralizing ADA (NAb), which inhibit the biological activity of a therapeutic protein by binding to the epitope(s) within or close to the active site(s) of the molecule or by causing conformational changes, thereby physically interfering with the ability of the drug to bind its target, Figure 1. Therefore, ADAs can alter a drug’s pharmacokinetic (PK) and pharmacodynamic (PD) properties, reducing drug efficacy. Overall, depending on the circulating ADA titers, a reduction in the drug concentration can be clinically significant. For example, in patients with low titers of ADAs, drug concentrations may remain high enough to be effective, while in patients who develop high titers of ADAs, a substantial portion of the drug will be neutralized and is likely to produce a clinical non-response over time [29,30,31].

### 3.2. TNF Inhibitors

TNFi is highly effective in attenuating inflammation and reducing symptoms in ARDs. Yet, a significant proportion of patients fail either primarily or secondarily due to drug inefficacy or adverse events. Currently, there are five FDA- and EMA-approved TNFi [32], which are described below, highlighting the prevalence of antibodies to the drug.

#### 3.2.1. Infliximab (IFX)

IFX is a chimeric anti-TNF mAb approved for SpA, Crohn’s Disease (CD), psoriasis (Ps), psoriatic arthritis (PsA), RA, and ulcerative colitis (UC) [33]. The development of immunogenicity to IFX has been reported in association with immune-mediated adverse events such as infusion reactions (due to the formation of ADAs). At least in RA, between 4 and 15% of patients treated with IFX could develop ADAs [34]. Nevertheless, a study based on the DANBIO registry (Danish nationwide registry) which included 218 RA patients-TNF-α naive that was followed by 52 weeks, showed that at weeks 6 and 14, 33 to 67% serum levels of INF-ADAs serum were detected, respectively. In addition, at the end of the follow-up (week 52), this percentage decreased to 54%, which could be due to minimal disease activity o remission [35]. The results between the reports can show significant differences between the percentages of ADAs, as shown in a systematic review where researchers point out that such differences are due to the lack of a standardized method and trials that consider quality criteria, which has not been established. That systematic review included 443 publications, of which 110 were related to IFX in different rheumatic diseases showing major differences in their results. The frequency of ADAs formation depends on the disease. For example, in RA, the frequency of ADAs-IFX varies from 8 to 62%, PsA 15–33%, JIA 26–42%, AS 6.1–69%, Ps 0–41%, CD 3–83% and UC 6–46%, then ranging from 0–83%. Although there is variability, the fact shows that the frequency of ADAs formation in patients treated with IFX can be low (0% in Ps) or exacerbated (83% in CD), but ADAs development is a real factor that must be taken in consideration because this can condition the success of the treatment [5].

#### 3.2.2. Adalimumab (ADL)

ADL is a fully human anti-TNF mAb IgG1 approved for ankylosing spondylitis (AS), Crohn’s Disease (CD), juvenile idiopathic arthritis (JIA), psoriatic arthritis (PsA), psoriasis (Ps), rheumatoid arthritis (RA), and urticarial vasculitis (UV). Several systematic reviews have reported the wide immunogenicity developed against ADL. In 2015, Arstikyte et al., carried out a study that included 143 patients that received biologics for a long term based on ACR criteria and then were evaluated for ADL-ADA production. They found that 4% of them developed ADAs with a mean concentration of 7.1 to 8.24 µg/mL [36]. Subsequently, in 2017 Strand et al., carried out a systematic literature review in MEDLINE, EMBASE, Cochrane Central Register of Controlled Trials, and Cochrane Database of Systematic Reviews. They searched around 32,584 publications, which included longitudinal observational studies (LOSs) to evaluate the frequency of ADL-ADAs development, but only 443 publications (394 studies) met the requirements. On the other hand, they mentioned that immunoassay methodologies, study design as well as therapeutic schemes could promote variability in the percentage of ADAs formation. Nonetheless, in 80 of the publications reviewed, they reported a frequency between 0–54% of the patients with ADAs formation [5].

#### 3.2.3. Golimumab (GLM)

GLM is also a fully human anti-TNF mAb IgG1, mainly approved for AS, PsA, and RA treatment [37,38]. In the same study carried out by Strand et al., (systematic literature review in MEDLINE, EMBASE, Cochrane Central Register of Controlled Trials, and Cochrane Database of Systematic Reviews) about the GLM-ADAs development, the results are heterogeneous and seem to depend on the disease that is being treated. For example, in AS, between 0–6.4% of patients analyzed developed GLM-ADAs, which can be considered a wide range of frequency. When we reviewed data to talk about PsA, the mean frequency was about 6%, a more punctual data than AS. However, in RA, the frequency rate changes again to a range between 2–10%. GLM is also indicated as another treatment in UC, and once again, the percentage of autoantibody formation increases, reaching 0–19%. As we can see, the frequency range data are non-homogeneous from one disease to another, from the ones GLM is prescribed [5].

#### 3.2.4. Etanercept (ETA)

ETA is a fusion protein between a human IgG1 Fc-tail and the TNF-receptor type 2 (TNFR2) approved for its clinical use in AS, JIA, PsA, Ps, and RA. Several reports have pointed out that this biological drug has a high capability to promote the development of ADAs. However, some scientific reports show that ETA cannot induce ADAs formation. Thomas et al., in 2015, conducted a systematic review and meta-analysis using the preferred reporting items for systematic reviews and meta-analyses (PRISMA) performing research within the databases PubMed, Web of Science, and the Cochrane Library. For RA, they reported that the mean for ETA-ADAs is only 3%, but this percentage represents only one within of the found articles because in others is 0%; however, they mentioned that in other autoimmune rheumatic diseases studied, the percentage remains to be 0%. When they divide this 3% among all diseases analyzed where ETA is used, they conclude that the average formation of ADAs is 1.2% [39]. Another report by Badder et al., in 2018 evaluated the ETA-ADAs development in 218 patients with JIA, and they mentioned that only 0.9% of the patients presented ETA-ADAs, but ADAs serum levels were only slightly above the lower limit of detection of the ELISA test, which was not significant for their study. In the same way, this idea was supported by another report where the serum values of ETA-ADAs in RA were 0% [40,41]. In contrast, the prevalence of ETA-ADAs in psoriasis reaches 18.3% [42,43]. Therefore, there is great variability in the mean percentages of ADAs, as reported by Strand et al., for example, ETA-ADAs serum levels in RA patients were found to be about 0–13%, 0% in PsA, 0–6% in JIA, 0% in AS and 2–5% in AS [5].

#### 3.2.5. Certolizumab Pegol (CZP)

CZP is a PEGylated Fab fragment of a humanized anti-TNF antibody approved for the treatment of CD, RA, Ps, and nonradiographic axial spondyloarthritis (nr-axSpA) [44,45]. CZP is indicated in pregnant women because it does not cross the placental barrier [46]. In addition, CZP has been reported to be among the biologic drugs that form fewer ADAs compared with IFX or ADL, although the levels of ADAs depend on the diseases for which such CZP is used. For example, in the study carried out by Strand et al., they found that between 3 to 32% of patients develop CZP-ADAs, mainly in RA [5]. In the study PRECiSE 3 with clinical trial number NCT00160524, 595 individuals were enrolled and received 400 mg of CZP daily for 7 years. It is worth mentioning that about 25% (150) of them developed CZP-ADAs, of which 94 had persistent CZP-ADAs. The rest of them had transient ADAs [47], which according to the mean proposed by Strand, however, is considered that trials to delve into its immunogenicity are scarce.

It has been proposed that the use of bDMARDs is one of the tools with the greatest potential for the treatment of most rheumatic inflammatory diseases, but the evaluation of the formation of ADAs is not within the parameters of periodic monitoring of the patient, which could be an indicator of the stage of the disease or the clinical response. In systematic reviews as well as in a longitudinal study focused on the exploration of the immunogenicity of TNFi across inflammatory diseases as well as its possible impact on efficacy/safety, the next frequencies of mean percentages of ADAs were presented: IFX (0–83%), ADL (0–54%), GLM (0–19%), ETA (0–18.3%) and CZP (3–37%) [5,42,47] Table 2. In addition, some authors have highlighted that IFX and ADL are the major triggers to promote immunogenicity, arguing that the frequency in each disease is due to possible mechanisms strongly related to subtherapeutic serum drug level and weak clinical response of the patients [48].

### 3.3. B Cell-Targeting Biologics

#### 3.3.1. Rituximab (RTX)

RTX is a chimeric murine/human mAb IgG-1/kappa isotype that recognizes the CD20 antigen present on B-lymphocytes promoting its depletion. RTX was first used in non-Hodgkin lymphoma treatment but is now used in combination with MTX to treat RA that had not responded to one or more types of treatment, including TNFi [68,69]. RTX treatment is associated with a high degree of ADAs, which correlates with the efficacy of B-cell depletion. In a cross-sectional study, the collection of serum samples from 339 MS patients immediately before a scheduled RTX infusion, ADAs were detected in 37% of relapsing/remitting MS and 26% in progressive forms of MS. There was a significant association between the presence and titers of ADAs and incomplete B-cell depletion [49]. In addition, in another clinical trial of 26 children treated with RTX for various immune-mediated diseases, six patients were ADA-positive (23.1%). In all ADA-positive patients, RTX concentrations were undetectable in contrast to ADA-negative patients, indicating that these ADA have a PK impact. Failure of B cell depletion was found in 5/6 ADA-positive and 1/19 ADA-negative patients, showing that these ADA have a PD impact. Moreover, in SLE patients, 50.0% (n = 4/8) had developed RTX-ADA [50]. Specific patient groups may be more prone to develop ADAs. For example, after the first RTX cycle, no AAV (ANCA-associated vasculitis) patients were ADA-positive compared to 37.8% of the SLE patients [70].

#### 3.3.2. Belimumab (BLM)

B-lymphocyte stimulator (BLyS), also known as the B-cell activating factor (BAFF), is a member of the TNF superfamily of cytokines. BLM is a fully human IgG1 targeting BLyS approved for the treatment of SLE [71]. In phase III BLISS-52 and BLISS-76, ADAs were detected in four patients receiving IV BLM 10 mg/kg. There was no formation of ADAs in patients receiving SC BLM 200 mg once a week in BLISS-SC. Currently, the clinical relevance of the presence of anti-BLM antibodies is unknown [51].

### 3.4. T Cell Activation Inhibitor/Co-Stimulation Modulator

#### Abatacept (ABT)

ABT is a recombinant and glycosylated fusion protein that has as its target the human cytotoxic T-lymphocyte-associated antigen 4 (CTLA-4 or CD152). This biological drug has an Fc-modified portion (hinge, CH2, and CH3 domains) of the human over the immune system. It is used in patients with RA, PsA, or pJIA who have not responded to one or more DMARDs, such as MTX or other biologic drugs [72,73].

During clinical trials (phase II and III), IV ABT administered with or without MTX demonstrated transient, low-titer immunogenicity without clinical consequence or influence on efficacy or safety. 2237 patients with pre- and post-baseline serum samples were eligible for assessment. Of these, 0.9–2.1% of patients presented anti-CTL-4 antibodies, and among 20 patients positive for anti-CTLA-4 reactivity, 13 were eligible for assessment with the neutralization bioassay, and within these, 8 patients showed neutralizing activity [52]. Whereas in a phase IIIb non-inferiority study, ABT-induced antibodies occurred in 1.1% of SC ABT-treated patients and 2.3% of IV ABT-treated patients [54]. A study designed to evaluate the immunogenicity of ABT, with or without MTX and in the absence of an initial IV loading dose of ABT (ACCOMPANY phase III study), showed that 3.9% (combination) and 4.1% (monotherapy) of patients developed transient immunogenicity, and no patients were antibody positive at month 4 [53].

### 3.5. IL-6R Inhibitors

IL-6 is a pleiotropic cytokine that regulates immune responses and inflammatory reactions and plays a pivotal role in the development of many chronic inflammatory diseases. IL-6R mAbs have been developed to reduce chronic IL-6-mediated inflammatory signaling and have primarily been employed for chronic inflammatory autoimmune conditions [74]. Currently, two IL-6 receptor antibodies are already available in the clinic and have shown benefits in patients with ARDs.

#### 3.5.1. Tocilizumab (TCZ)

TCZ is a recombinant humanized mAb, and its target molecule is the IL6 receptor (IL-6R) approved for RA, systemic juvenile idiopathic arthritis (sJIA), or polyarticular course juvenile idiopathic arthritis (pJIA). Clinical experience with TCZ, both IV and SC administration, has demonstrated low immunogenicity in several indications, including RA, Castleman’s disease, and JIA [75]. Anti-TCZ antibodies developed among the small proportion of patients had no evident impact on PK, efficacy, or safety. ADAs were identified in 47 of 3094 (1.5%) and 69 of 5806 (1.2%) patients treated with TCZ in SC or IV, respectively, whereas NAbs were confirmed in 40 of 3094 (1.3%) and 54 of 5806 (0.9%) of these patients, respectively. ADA development was also comparable between patients who received TCZ monotherapy and those who received concomitant csDMARDs (0.7–2.0%) [55]. Similar data are reported in the SUMMACTA study to compare the efficacy and safety of SC versus IV formulations of TCZ in patients with RA with an inadequate response to DMARDs. Only five patients in each group were positive (0.8% and 0.8%) for both the confirmation and neutralizing assays post-baseline [56].

#### 3.5.2. Sarilumab (SLM)

SLM is an alternative fully mAb to IL-6R; it has also demonstrated efficacy and safety as well as it has been approved for the treatment of RA. It has a greater affinity for the IL-6R and a longer half-life than TCZ [74,76]. A recent report describes the immunogenicity profile of SLM in Japanese patients with RA—KAKEHASI and HARUKA studies. Positive ADA assay responses occurred in 10/149 (7.1%) patients treated with SLM 150 mg and 13/185 (7.0%) patients treated with SLM 200 mg, with persistent responses in 2 (1.4%) and 4 (2.2%) patients, respectively. Peak ADA titer was 30. No patients treated with the 150 mg dose and one patient (0.5%) treated with the 200 mg dose exhibited Nabs [57]. Similar results were previously observed in a study conducted in predominantly Caucasian populations, where persistent ADAs occurred in 8 patients (12.3%) receiving SLM 150 mg every 2 weeks, seven of whom (10.8%) had NAbs, and in 4 patients (6.1%) receiving SLM 200 mg every 2 weeks, two of whom (3.0%) had NAbs; all exhibited low antibody titers [58]. Although SLM is a fully human mAb and TCZ is a humanized mAb, both ADA titers were low and persistent ADAs, and NAbs occurred in rare frequency.

### 3.6. IL-17A Inhibitors

The relevance of IL-17 in rheumatic diseases has been demonstrated with the approval of three different inhibitors of the IL-17 signaling for the treatment of PsA, plaque psoriasis (PsO), and AS [59].

#### 3.6.1. Secukinumab (SCK)

SCK is a fully human IgG1/kappa mAb against IL-17A indicated for the treatment of PsO and PsA in adult patients who have shown an inadequate response to previous treatments with DMARDs, axial spondylarthritis (AxSpA), AS, and nr-AxSpA. SCK has shown low immunogenicity in several studies (less than 1%), in human in vitro assays [77], in psoriasis patients treated up to 5 years [78], in patients with moderate-to-severe PsO [79], and in patients with PsA and AS exposed for up to 52 weeks with a low (<1%) incidence of immunogenicity [60]. SCK has demonstrated significantly lower immunogenicity (0.4%) potential compared to ixekizumab in anti-drug-related T-cell responses in vitro assay [61]. This could be explained because ixekizumab is a humanized anti-IL-17A mAb. However, it was generally well tolerated and exhibited low immunogenicity in trials during up to 60 weeks of therapy in patients with PsO [80]. Finally, in a systematic review conducted to explore the immunogenicity of several biological agents across inflammatory diseases, overall rates for ADAs for SCK were 0–1%, like these reports [5].

#### 3.6.2. Ixekizumab (IXK)

IXK is a humanized monoclonal antibody (IgG4) directed against IL-17A and IL-17A/F. It is currently approved for moderate to severe PsO in adult candidates for systemic treatments and active PsA in adult patients with an insufficient response or intolerance to one or more DMARDs. IXK is generally well tolerated and showed low immunogenicity in the UNCOVER trials during up to 60 weeks of therapy [80]. ADAs against IXK were developed in 103 of 1150 patients (9.0%) in the 2-wk dosing group on the three UNCOVER trials during the induction period; 19 patients (1.7%) had high titers of ADAs (≥1: 1280), accompanied by a lower clinical response than that of patients who had none or low-to-moderate titers of ADAs [62].

#### 3.6.3. Brodalumab (BDL)

BDL is a human, anti-IL17 receptor mAb IgG2 that binds with high affinity to human IL-17 receptor A and blocks the biological activity of the pro-inflammatory cytokines IL-17A and IL-17F, resulting in the inhibition of inflammatory and clinical symptoms associated with psoriasis and is approved for PsO [81,82,83]. Data from phases 1, 2, and 3 of the studies of BDL in psoriasis shows that 2.7% of patients had a positive test for binding ADAs after receiving BDL; ADAs were transient in 1.4% of patients, and there were no NAbs. In addition, of all patients who experienced the persistence of the disease and were retreated with BDL (210 mg every 2 weeks), none were ADA positive. Therefore, BDL compares favorably with other biologics in terms of immunogenicity and high rates of efficacy recapture upon retreatment [63].

### 3.7. IL-12/23 and IL-23p19 Inhibitors

#### 3.7.1. Ustekinumab (UTK)

UTK is a fully human mAb to IL-12 and IL-23 receptor (IL-12R/IL-23R) to block the intracellular signaling avoiding inflammatory events and T cell activation. It is used in patients with PsO, PsA, CD, and UC [84,85]. Following induction/maintenance treatment in the UNITI/IM-UNITI studies of UTK for Crohn’s disease, the rates of antibodies to UTK formation (using a sensitive drug-tolerant assay) remained low through three years, with 4.6% (11/237) [86]. Similar results were previously shown at two years, with 4.2% (10/237) of UTK-treated patients that tested positive for antibodies to UTK [87], and at 72 weeks with 5.2%, in a phase III study (PHOENIX 1) patients with moderate-to-severe psoriasis [88]. Overall, the prevalence of ADAs against UTK in patients with psoriasis was reported in 3.8–6% of patients. Two of six studies reported that anti-UTK antibodies were associated with lower psoriasis area and severity index responses, and three UTK studies noted that most of these antibodies were neutralizing (PHOENIX 2), but the range was not reported [42]. Furthermore, in another systematic review conducted to explore the immunogenicity of several biological agents across inflammatory diseases, overall rates for ADAs for UTK were 1–11%, like these reports [5].

#### 3.7.2. Guselkumab (GKM), Risankizumab (RZM) and Tildrakizumab (TZM)

Newer IL-23p19 antagonists have been produced and applied in clinics that show a translational revolution in the treatment and management of psoriasis [89]. Recently, a systematic review reported the incidence of ADAs with the IL-23 inhibitors and was as follows: 4.1–14.7% with GKM, 14.1–31% with RZM, and 6.51–18% with TZM. The incidence of NAbs ranged between 0.1–0.6% with GKM, 2.1–16% with RZM, and 2.5 to 3.2% with TZM. There was no evidence of reduced efficacy of psoriasis treatment with ADA presence alone. However, some studies found a reduction in clinical response with high ADA titers or with the presence of NAbs [64].

### 3.8. IL-1 Inhibitors

IL-1 receptor antagonist (IL-1Ra) is an endogenous, competitive antagonist of the IL-1 receptor, which modulates the biological actions of IL-1 by preventing signal transduction. There are now agents available on the market capable of blocking IL-1 [90].

#### 3.8.1. Anakinra (ANA)

ANA is a recombinant form of human IL-1Ra. It is structured by a human nonglycosylated recombinant fusion protein IL-1Ra and has added an extra methionine residue at the amino terminus also, nullifying its inflammatory properties. ANA is a competitive antagonist of IL-1 that prevents the link of IL-1α and IL-1β with IL-1R and is used in patients with AR [90,91,92]. Currently, ANA is used only marginally for the treatment of RA. This has limited the experience with this biological product in RA, with a lack of data from long-term observational studies or registries. However, immunogenicity is the most frequent adverse event but with a low frequency (<1%) related to ANA administration [65,66].

#### 3.8.2. Canakinumab (CKM)

CKM is a fully human mAb anti-IL-1β that belongs to the IgG1/kappa isotype; it binds to the IL-1 beta (IL-1β); its purpose is to prevent its link with IL-1R avoid the inflammatory effects of IL-1 and is used to treat AR and sJIA [90,93,94]. A low immunogenicity incidence of 3.1% was seen in patients with sJIA, and none of the patients had NAbs [67]. Like ANA, little evidence of CKM immunogenicity is available because clinical studies focus on the safety (adverse events) but not the assessment of ADAs [95,96,97].

In summary, the proportions of ADAs among the biological agents used in ARDs are shown in Table 2. The lowest proportions of ADA-positive were observed for ANA or SCK (less than 1%) and the highest with IFX, ADL, or RTX. These could be explained in part because TNFi has been available for the treatment of ARDs for the last 20 years and still accounts for a substantial proportion of drugs used to treat autoimmune diseases, whereas since RTX was approved, it’s been widely used for RA. However, there is little knowledge of newer biological products in ARDs, with a lack of data from long-term observational studies or registries. Moreover, NAb-positive patients NAbs are not usually reported.

## 4. ADAs Detection and Clinical Implications

### 4.1. Assays for ADAs Detection

Although the humanization of mAbs has reduced the immunogenicity of biological agents, all protein therapeutics, including fully human mAbs, can provoke immunogenicity, most notably the development of ADAs. The neutralizing ability of antibodies present in positive samples needs to be evaluated as part of the immunogenicity assessment since this often correlates with diminished clinical responses to biological products. A sample designated for ADA testing is first screened and confirmed for the presence of binding antibodies. Then, only positive samples are tested in a NAb assay. Screening assays should be sensitive, specific, and validated methods for assessing immunological responses. These methods should be able to detect all isotypes of antibodies against biological agents. The available analytical assays include enzyme-linked immunosorbent assay (ELISA), radioimmune assay (RIA), or electrochemiluminescence (ECL). However, the detection and measurement of ADA are complex, and the results can be affected by the assay used [98].

Two types of NAb assays are mainly used—cell-based assays and non-cell-based assays. EMA and FDA immunogenicity guidance documents show a preference for cell-based NAb assays due to their physiological relevance because NAbs can trigger clinical effects. Therefore, specific and sensitive in vitro methods are expected [9,10]. These cells-based NAb assays are especially relevant for therapeutic proteins that resemble an endogenous protein where cross-reactive NAbs could result in an autoimmune state, thereby affecting patient safety. At the same time, a non-cell-based assay appears to be acceptable when a drug’s mechanism of action is to solely neutralize a soluble target [99,100].

### 4.2. Clinical Implications and Therapeutic Monitoring

It is well known that the presence of ADAs is associated with the ability to decrease drug levels and contribute to clinical inefficacy. This could be of concern because many factors potentially related to biological therapy as well as clinical aspects of the patient must be considered, making it difficult for the physician to prescribe an appropriate drug therapy regimen the first time [39,101].

In addition, the presence of ADAs is also associated with AE, such as infusion reactions. These reactions are the most common ADA-related AE described for TNFi, and they are characterized by symptoms such as fever, pruritus, bronchospasm, or cardiovascular collapse during or within the first day after drug administration. Infusion-related reactions can range from mild (e.g., rash, pruritus, dizziness, dyspnea) to severe (anaphylactic-like reactions such as hypotension and respiratory distress) [31].

Moreover, the clinical effects of the formation of ADA depend on the amount of drug neutralized by ADA and the amount of free drug present. In this context, the therapeutic drug monitoring (TDM) of biopharmaceuticals could be helpful in several clinical situations, such as loss of response to therapy, the interpretation of side effects to therapy, and determining dose adjustments. It is important to mention that the TDM not only refers to the principle of using biopharmaceutical blood concentration for therapeutic decisions but also includes—although optionally—ADAs determination. Although validated TDM assays are widely available, there are no adequate guidelines on whether and how to use them in clinical practice. Therefore, EULAR proposed the use of a conglomerate of points together with the TDM assay to obtain accurate information on the serum concentration of the biopharmaceutical, the formation of ADA, the optimization of the biopharmaceutical dose, the reduction of adverse events, or the potential impact of ADA on biologic therapy over time [102]. Two EULAR task forces have agreed to define precise aspects for when it is necessary to evaluate the development of ADA and whether it affects the serum concentration of the biopharmaceutical as well as the outcome of therapy [103].

Accordingly, EULAR proposed the use of six overarching principles and 13 points to consider TDM in inflammatory rheumatic and musculoskeletal diseases. The points to consider highlighting the clinical utility of the measurement and interpretation of biopharmaceutical blood concentrations and ADAs in specific clinical scenarios, including factors that influence these parameters. The first point to consider is that the determination of serum levels of biopharmaceuticals must be performed in a validated laboratory. Since the most widely used method for the determination of biopharmaceuticals is the ELISA type, which gives different results but has a good correlation, the information on the correlation of the results obtained with ELISA compared to a homogenous mobility shift assay (MSA), immunofluorometric assay or a reporter gene assay (RGA) is still insufficient. Results are reported in different detection units (e.g., ug/L, AU/mL). Because of these differences, it is suggested that assays need to be standardized according to international practice [104,105,106,107]. The second point is that the measurement of ADA must be performed with an assay that is consistent, stable, and easily reproducible over time. The determination of ADA is complicated because of other factors that may affect its detection. For example, ADA has been reported to interact with other molecules, such as a rheumatoid factor. On the other hand, current tests for the determination of ADA are aimed at scientific research but not yet at clinical practice. For this reason, the determination of ADA, which should be considered in clinical practice, should be performed in conjunction with the biopharmaceutical in a blood sample. Moreover, the presence of ADA in clinical practice could only be relevant if the concentration of the biopharmaceutical in the blood sample is lower or absent [102,107]. The third point highlighted by EULAR is that the concentration of the biopharmaceutical in the blood depends on the dose, the administration interval, and the date of the last dose. It is recommended that blood sampling for biopharmaceutical blood concentration (and ADAs) measurements should preferably be performed prior to the next administration when concentrations are at, or close to, trough. This is especially important and easier to operationalize with intravenous dosing. For subcutaneous dosing, trough measurement is less critical. Nonetheless, the point recommends measurement as close as possible to the next administration. In addition, this point mentions that when interpreting biopharmaceutical blood concentrations and based on the PK properties of biopharmaceuticals, the task force advises considering dose, administration interval and route of administration, date of last dose, and, especially in the case of therapeutic mAbs, co-administration of MTX. In this context, it is clear that the time for determining the concentration of the biopharmaceutical in the blood sample and its quantification could be influenced by certain characteristics of the patient that could affect PK, such as body weight, co-treatments, disease activity, and treatment compliance [107,108].

Besides, the next point mentions that despite an association with clinical response, the use of biopharmaceutical blood concentrations to guide dosing is not recommended due to the lack of an identified optimal range for most biopharmaceuticals in most indications. A number of studies have attempted to identify a therapeutic range for each biopharmaceutical indication. However, due to large variations in blood concentrations, different clinical outcome measures between studies, and differences in the timing of blood sampling in relation to dosing, no clear optimal therapeutic range could be identified in most cases. Even routine use of proactive TDM is not recommended in the management of inflammatory rheumatic and musculoskeletal diseases. On the other hand, the task force agreed that the measurement of biopharmaceutical blood concentrations up to 3 months after the commencement of treatment could be considered to predict future efficacy. This is one of the most important points to consider because mAbs used as therapeutic proteins have different PK characteristics due to their particular physical characteristics, generally showing a smaller volume of distribution and a longer half-life, and their clearance is related to protein degradation and target binding. Another point to consider for TDM of biopharmaceuticals is that the presence of ADA can provide insight into the reasons for clinical non-response to treatment. However, there is contradictory evidence that the presence of ADA in a first TNFi predicts a better treatment response to a second TNFi. Switching from an immunogenic to a less immunogenic TNFi might be considered, as patients might also be more likely to develop ADA to a second immunogenic TNFi [107]. However, intra-class and inter-class switches are both effective options in patients who fail the first biologics. In patients who develop secondary failure to the first biologics due to ADA, a second biological drug within the same class may usually be considered [109,110,111,112].

## 5. Conclusions

All protein therapeutics, including bDMARDs, can induce immunogenicity, especially the development of ADAs. The biological and clinical effects caused by ADA depend on the amount of the neutralized and free drug. In this context, it is important to report the amount of ADA in patients in addition to the percentage of patients who develop ADAs. However, several studies have shown that ADAs occur in low frequency and low titer and do not affect the safety or efficacy of biologics. It is noteworthy that most of these studies were sponsored by pharmaceutical companies. This may be in part because information on immune responses is part of the product development program, and immunogenicity issues should be further addressed in the risk management plan as part of the marketing authorization application in accordance with current legislation and pharmacovigilance guidelines. Nonetheless, the immunogenicity potential of biologics agents should be considered an important part of clinical practice, even though it generally occurs infrequently, not only because ADAs interfere with the efficacy of biologics but also because they increase the risk of other adverse events on treatment, including infusion reactions.

The development of next-generation bDMARDs and the expansion of their therapeutic indications to other autoimmune diseases, as well as new therapies with small molecules such as Janus kinase inhibitors (JAK) (tsDMADRs), are expected.

## Figures and Tables

**Table 1:** Biologic agents licensed or approved for the treatment of ARDs by target [9,10].

Target	Biological Agent	Format	Indication
TNF-α	Infliximab	Chimeric mAb	CD, UC, PsA, PsO, AS, RA
TNF-α	Adalimumab	Fully human mAb	RA, PsA, PsO, AS, CD, JIA, UV
TNF-α	Golimumab	Fully human mAb	AS, RA, PsA
TNF-α	Etanercept	Fusion protein	RA, pJIA, PsA, AS, PsO
TNF-α	Certolizumab pegol	Humanized mAb	CD, RA, PsA, AS, nr-axSpA
CD20	Rituximab	Chimeric mAb	RA, SLE
BLyS	Belimumab	Fully human mAb	SLE
CTLA-4	Abatacept	Fusion protein	RA, PsA
IL-6R	Tocilizumab	Humanized mAb	RA, sJIA, pJIA
IL-6R	Sarilumab	Fully human mAb	RA
IL-17A	Secukinumab	Fully human mAb	AS, RA, PsA, PsO, uveitis, AxSpA, nr-AxSpA
IL-17A	Ixekizumab	Humanized mAb	PsO, PsA, and AS
IL-17RA	Brodalumab	Fully human mAb	PsA
IL-12R/IL-23R	Ustekinumab	Human mAb	PsO, CD, UC
IL-23 p19	Risankizumab	Humanized mAb	PsO
IL-23 p19	Tildrakizumab	Humanized mAb	PsO
IL-23 p19	Guselkumab	Fully human mAb	PsO
IL-1RA	Anakinra	Fusion protein	RA and autoinflammatory fever syndrome
IL1-β	Canakinumab	Fully human mAb	AR, sJIA

Legend: AS: ankylosing spondylitis; AxSpA: axial spondylarthritis; BLyS: B-lymphocyte stimulator; CTLA-4: Cytotoxic T-Lymphocyte Antigen 4; CD: Crohn’s Disease; sJIA: systemic juvenile idiopathic arthritis; pJIA: polyarticular course juvenile idiopathic arthritis; mAb: monoclonal antibody; MTX, methotrexate; nr-axSpA: non-radiographic axial spondylarthritis; PsA: psoriatic arthritis; PsO: plaque psoriasis; RA: rheumatoid arthritis; SLE: systemic lupus erythematosus; TNF: tumor necrosis factor; UC: Ulcerative Colitis; UV: Urticarial Vasculitis.

**Table 2:** Biological agents and rates of anti-drug antibodies (ADAs) and neutralizing anti-drug antibodies (NAbs) reported in ARDs.

	Biological Agent	Abbreviation	ADAs% Min–Max	Nabs% Min–Max	Ref
TNFi	Infliximab	IFX	0–83.0	Not reported	[5,42]
	Adalimumab	ADL	0–54.0	Not reported	[5,42]
	Golimumab	GLM	0–19.0	Not reported	[5]
	Etanercept	ETA	0–18.3	Not reported	[5,42]
	Certolizumab pegol	CZP	3–37.0	Not reported	[5]
B cell-targeting biologics	Rituximab	RTX	23.1–50.0	Not reported	[49,50]
	Belimumab	BLM	0–4.8	Not reported	[51]
T cell activation inhibitor/co-stimulation modulator	Abatacept	ABT	0.9–4.1	0–0.4	[52,53,54]
IL-6R inhibitors	Tocilizumab	TCZ	0.7–2.0	0.8–1.3	[55,56]
	Sarilumab	SLM	1.4–12.3	0–10.8	[57,58]
IL-17A inhibitors	Secukinumab	SCK	0–1.0	Not reported	[5,59,60,61]
	Ixekizumab	IXK	1.7–9.0	Not reported	[62]
	Brodalumab	BDL	1.4–2.7	0	[59,63]
IL-12/23 and IL-23p19 inhibitors	Ustekinumab	UTK	1.0–11.0	Not reported	[5,42]
	Guselkumab	GKM	4.1–14.7	0.1–0.6	[64]
	Risankizumab	RZM	14.1–31.0	2.1–16.0	[64]
	Tildrakizumab	TZM	6.51–18.0	2.5–3.2	[64]
IL-1 inhibitors	Anakinra	ANA	<1	Not reported	[65,66]
	Canakinumab	CKM	3.1	0	[67]

## Data Availability

Not applicable.
